# Functional analysis of filipin tailoring genes from *Streptomyces filipinensis* reveals alternative routes in filipin III biosynthesis and yields bioactive derivatives

**DOI:** 10.1186/s12934-015-0307-4

**Published:** 2015-08-07

**Authors:** Tamara D Payero, Cláudia M Vicente, Ángel Rumbero, Eva G Barreales, Javier Santos-Aberturas, Antonio de Pedro, Jesús F Aparicio

**Affiliations:** Area of Microbiology, Faculty of Biology, Universidad de León, 24071 León, Spain; Institute of Biotechnology INBIOTEC, Parque Científico de León, Avda. Real, no 1, 24006 León, Spain; Department of Organic Chemistry, Faculty of Sciences, Campus de Cantoblanco, Universidad Autónoma de Madrid, 28049 Madrid, Spain; Department of Molecular Microbiology, John Innes Centre, Norwich Research Park, Norwich, NR4 7UH UK

**Keywords:** Antifungal agent, Cytochrome P450 monooxygenase, Filipin biosynthesis, Filipin derivatives, Polyene macrolide, *Streptomyces*

## Abstract

**Background:**

*Streptomyces filipinensis* is the industrial producer of filipin, a pentaene macrolide, archetype of non-glycosylated polyenes, and widely used for the detection and the quantitation of cholesterol in biological membranes and as a tool for the diagnosis of Niemann–Pick type C disease. Genetic manipulations of polyene biosynthetic pathways have proven useful for the discovery of products with improved properties. Here, we describe the late biosynthetic steps for filipin III biosynthesis and strategies for the generation of bioactive filipin III derivatives at high yield.

**Results:**

A region of 13,778 base pairs of DNA from the *S. filipinensis* genome was isolated, sequenced, and characterized. Nine complete genes and two truncated ORFs were located. Disruption of genes proved that this genomic region is part of the biosynthetic cluster for the 28-membered ring of the polyene macrolide filipin. This set of genes includes two cytochrome P450 monooxygenase encoding genes, *filC* and *filD*, which are proposed to catalyse specific hydroxylations of the macrolide ring at C26 and C1′ respectively. Gene deletion and complementation experiments provided evidence for their role during filipin III biosynthesis. Filipin III derivatives were accumulated by the recombinant mutants at high yield. These have been characterized by mass spectrometry and nuclear magnetic resonance following high-performance liquid chromatography purification thus revealing the post-polyketide steps during polyene biosynthesis. Two alternative routes lead to the formation of filipin III from the initial product of polyketide synthase chain assembly and cyclization filipin I, one trough filipin II, and the other one trough 1′-hydroxyfilipin I, all filipin III intermediates being biologically active. Moreover, minimal inhibitory concentration values against *Candida utilis* and *Saccharomyces cerevisiae* were obtained for all filipin derivatives, finding that 1′-hydroxyfilipin and especially filipin II show remarkably enhanced antifungal bioactivity. Complete nuclear magnetic resonance assignments have been obtained for the first time for 1′-hydroxyfilipin I.

**Conclusions:**

This report reveals the existence of two alternative routes for filipin III formation and opens new possibilities for the generation of biologically active filipin derivatives at high yield and with improved properties.

**Electronic supplementary material:**

The online version of this article (doi:10.1186/s12934-015-0307-4) contains supplementary material, which is available to authorized users.

## Background

Filipin is a 28-membered ring pentaene macrolide antifungal antibiotic produced by *S. filipinensis*, *S. avermitilis*, and *S. miharaensis* [1–3]. Unlike the majority of polyenes, it is devoid of sugar, and constitutes the archetype of non-glycosylated polyenes. However, it also interacts with membrane sterols causing the alteration of membrane structure [4]. Most polyene macrolides display significantly higher affinity for ergosterol (the main sterol in fungal membranes) than for cholesterol-containing membranes (the sterol in mammalian cells), and this is thought to be the reason for the selective toxicity of these molecules, but filipin shows a similar affinity for both sterols. This property makes it useless for its application in human therapy due to its toxic side effects, but has permitted its application as a tool for the diagnosis of Niemann–Pick type C disease, a characteristic cholesterol overloaded lysosomal disorder of genetic origin [5], and as a probe for the detection and quantification of cholesterol in cellular membranes [6]. As other macrocyclic polyketides, filipin is synthesized by the action of type I modular polyketide synthases. Its biosynthetic gene cluster (*pte*) has been identified in the avermectin-producing *S. avermitilis* NRRL 8165 upon sequencing of its genome and encodes 14 polyketide synthase modules within five multifunctional enzymes, and eight additional proteins that presumably govern modification of the polyketide skeleton and regulation of gene expression [7–9].

The post-polyketide synthase biosynthetic tailoring of polyene macrolides usually involves oxidations catalysed by cytochrome P450 monooxygenases (P450s or CYPs). Members from this class of enzymes are common in macrolide biosynthetic gene clusters, and their specificities vary considerably toward the substrates utilised and the positions of the hydroxyl functions introduced. In nature, filipin is produced as a mixture of related compounds known as the filipin complex (filipins I–IV) [10], being filipin III the major component. These components vary in the number of post-polyketide hydroxyl functions introduced, thus filipin I has two hydroxyl groups fewer than filipin III, filipin II is filipin I hydroxylated at C26 [11] and filipin III the result of filipin II hydroxylation at C1′, while filipin IV is thought to be an epimer of filipin III [12]. Of these compounds, only filipin III has been structurally characterised [13].

Two cytochrome P450 monooxygenases have been proposed to be responsible for the variability of the filipin complex in *S. avermitilis*, CYP105P1 (PteC) and CYP105D6 (PteD), and their crystal structures and in vitro catalytic activities have been determined [14]. Although their role in vivo remains uncharacterized, CYP105P1 and CYP105D6 catalyze filipin hydroxylation in vitro at C26 and C1′ respectively [14].

In order to gain insight into the mechanism of filipin III formation we set out to get gene deletion mutants of both cytochrome P450 monooxygenases, but *S. avermitilis* turned out to be a very low producer [9], hence we decided to investigate filipin biosynthesis in *S. filipinensis*, a strain that produces 1,000-fold more. Interestingly, we found that two alternative routes lead to the formation of filipin III from the product of polyketide synthase assembly and cyclisation filipin I.

## Results

### Identification and cloning of filipin tailoring genes

The filipin biosynthetic genes were identified by hybridization using a probe obtained by PCR amplification of *S. filipinensis* chromosomal DNA with oligonucleotides derived from conserved stretches of the filipin PAS-LuxR regulator *pteF* from *S. avermitilis* (see “Methods”). A cosmid library was constructed and a total of 13,778 bp of contiguous DNA was cloned. All the cosmids were mapped with restriction enzymes *Not*I, *Bam*HI and *Eco*RI. Internal *Not*I fragments were the same size as their homologous fragments of *S. filipinensis* total DNA, suggesting that the cloned DNA was not rearranged.

**Fig. 1:**
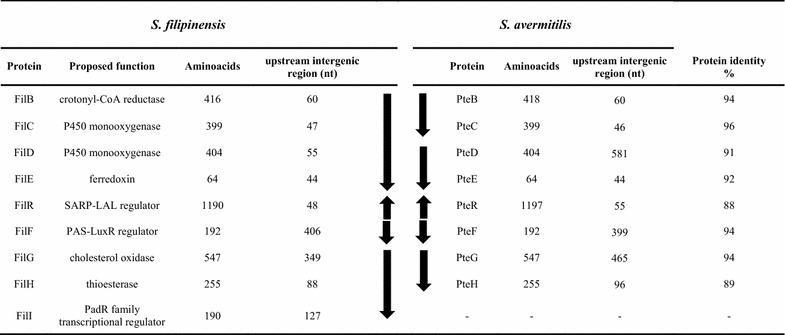
Comparison of filipin tailoring gene clusters from *S. filipinensis* and *S. avermitilis*. The size of the upstream region of each gene is indicated in nucleotides. *Arrows* indicate the organization of transcriptional units. The transcriptional organization of *S. avermitilis* has been included for comparison [9].

Once a gene homologous to *pteF* was identified (*filF*), the remaining genes of the subcluster were identified by chromosome walking. The deduced gene organization within this region is shown in Fig. 1.

### In silico analysis and arrangement of genes

Computer-assisted analysis of the sequenced region revealed nine complete genes, and two truncated open reading frames (ORFs). All these genes, but one (see below), showed a good synteny with the homologous *pte* genes from the filipin cluster of *S. avermitilis* [8], and following their nomenclature they were named *filB*, *filC*, *filD*, *filE*, *filR*, *filF*, *filG*, and *filH.* The degree of identity among coding regions ranged between 87 and 96% (at the protein level; Fig. 1) with a degree of divergence around 10%, whereas among intergenic regions such variability was much higher reaching a 40% divergence, including nucleotide deletions, substitutions, and insertions. Some intergenic regions were shorter in *S. filipinensis* (Fig. 1). For instance, in the region between *filC* and *filD* a 526 nt deletion occurred, while in the region between *filF* and *filG* there is a deletion of 116 nt (Fig. 1). Changes in the intergenic regions could potentially have effects on the gene transcription given that these regions may include promoter activities (see below).

Upstream from this set of genes (Fig. 1) there is an incomplete ORF whose deduced product showed high identity with PteA5 from *S. avermitilis*, the last polyketide synthase during filipin biosynthesis [2]. The product of the gene located downstream, *filB*, is a putative 2-octenoyl-CoA reductase that may provide the hexylmalonyl-CoA needed for the last polyketide chain extension during filipin biosynthesis [15]. The two genes that follow (*filC* and *filD*) encode cytochrome P450 monooxygenases (399 and 404 amino acids respectively) that by analogy with their counterparts from *S. avermitilis* are presumed to be responsible for hydroxylations at C26 and C1′ [14]. The *filE* gene immediately downstream from *filD* (Fig. 1) encodes a small acidic protein (64 amino acids) with convincing similarity to ferredoxins.

Convergent to *filE* lays *filR*. FilR (1,190 aa) is orthologous to the transcriptional activator of pimaricin biosynthesis PimR, an archetype of a particular class of regulators that combine an N-terminal DNA-binding domain corresponding to the SARP family of transcriptional activators with a C-terminal half homologous to guanylate cyclases and LAL regulators (Large ATP-binding regulators of the LuxR family) [16]. Recently we have characterized PimR mode of action, and determined that it binds an operator that contains three heptameric direct repeats of the consensus CGGCAAG with 4 bp spacers upstream from cognate promoters [17]. Divergently transcribed from *filR* is *filF*, a regulatory gene encoding a PAS-LuxR regulator (192 amino acids). These type of regulators are characteristic of polyene gene clusters [18], and have been demonstrated to be involved in the direct activation of given promoters within the cluster [19]. Recently, we have characterized PteF mode of action and determined that it activates transcription from the promoters of polyketide synthase genes *pteA1* and *pteA2* directly [9], and cross-regulates other clusters, e.g. the one involved in oligomycin biosynthesis [20].

The cluster also contains a cholesterol oxidase encoding gene, *filG* (Fig. 1), whose product has been proposed to act as antifungal sensor in *S. natalensis* [21], and a gene, *filH*, whose product resembles discrete thioesterases involved in polyketide proofreading [22, 23].

**Fig. 2:**
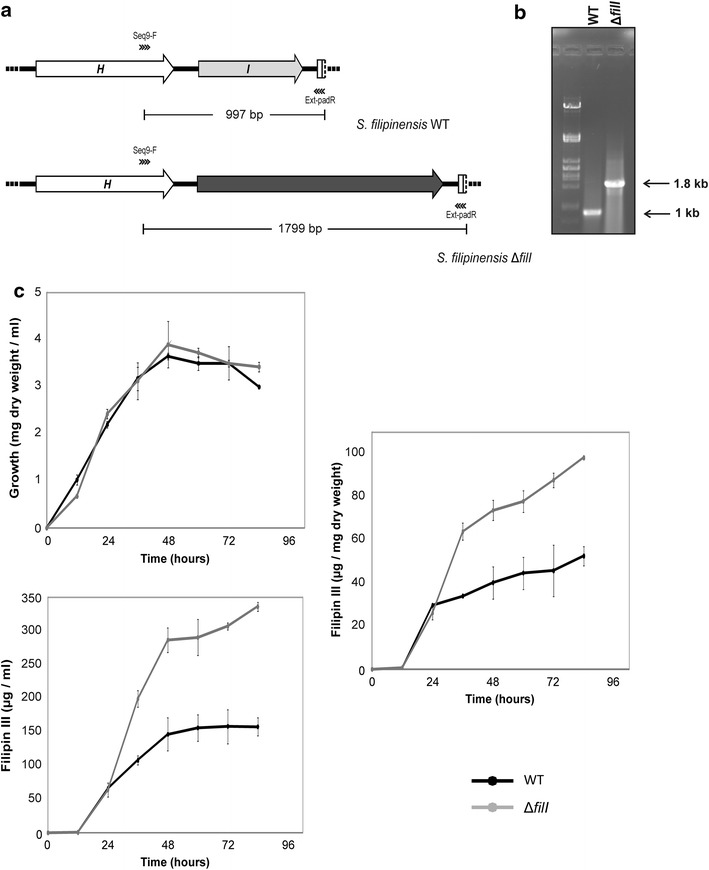
FilI inactivation increases filipin III production. **a** Predicted PCR fragment amplification of the parental strain and the mutant. The primers used in the assay are indicated with *arrowheads*. The acc(3)IV-oriT cassette is indicated in *black*. **b** PCR analysis of the wild type and the mutant. **c** Growth curves and time course quantification of the filipin III production in *S. filipinensis* (*black*) and *S. filipinensis ΔfilI* (*grey*).

Downstream from *filH*, there is an ORF that is absent in *S. avermitilis* which shows a putative dipeptidase gene (SAV 7575) instead. Its product (FilI) showed convincing similarity to PadR transcriptional regulators (Pfam PF03551), a class of regulators that control diverse processes including multi-drug resistance, virulence and detoxification, and secondary metabolite biosynthesis [24–26]. To ascertain that this gene belonged to the filipin cluster, we inactivated it by means of PCR targeting (see “Methods”). The fermentation broth, produced by double cross-over mutants, was extracted with ethyl acetate and analysed for the presence of filipin III (the major component of the filipin complex). HPLC assays indicated that filipin III production by the mutant strain was severely increased when compared with the parental strain, while showing a similar growth profile (Fig. 2). *S. filipinensisΔfilI* produced about double of the filipin III accumulated by the wild-type strain at 72 h (280 mg/l) (Fig. 2). This result indicates that *filI* belongs to the cluster and that its product FilI must be a negative regulator of filipin biosynthesis (Fig. 2). Flanking the right end of the cluster, and downstream from *filI*, there is an incomplete ORF whose partial product showed convincing similarity to acyl-CoA dehydrogenases.

### Organization of *fil* transcriptional units

In order to define an overall picture of the transcriptional arrangement of the *fil* genes, an accurate identification of operons was needed. We thus decided to analyse the possible co-transcription of neighbouring genes by reverse transcriptase-polymerase chain reaction experiments. Total RNA was prepared from *S. filipinensis* after growth for 48 h. Primers were designed to obtain cDNAs corresponding to unabated transcription between two genes (Additional file 1: Table S1). Transcripts were analysed after 40 PCR cycles to ensure that even low-level transcribed genes were detected. A primer pair designed to amplify a cDNA of the *rrnA1* gene was used as an internal control. These analyses were carried out at least three times for each primer pair. Following this strategy, *filA5*, *filB*, *filC*, *filD*, and *filE* could be co-transcribed since unabated transcription was observed between the upstream and the downstream gene. Similarly *filG*, *filH and filI* could also be co-transcribed. No transcripts were detected connecting *filF* and *filG*, thus suggesting that *filG* should have its own promoter. The genes *filR* and *filF* must also have their own promoters as can be deduced from their chromosomal arrangement in a divergent manner. Figure 1 shows the deduced organization of transcriptional units.

### Functional analyses of P450 monooxygenases

**Fig. 3:**
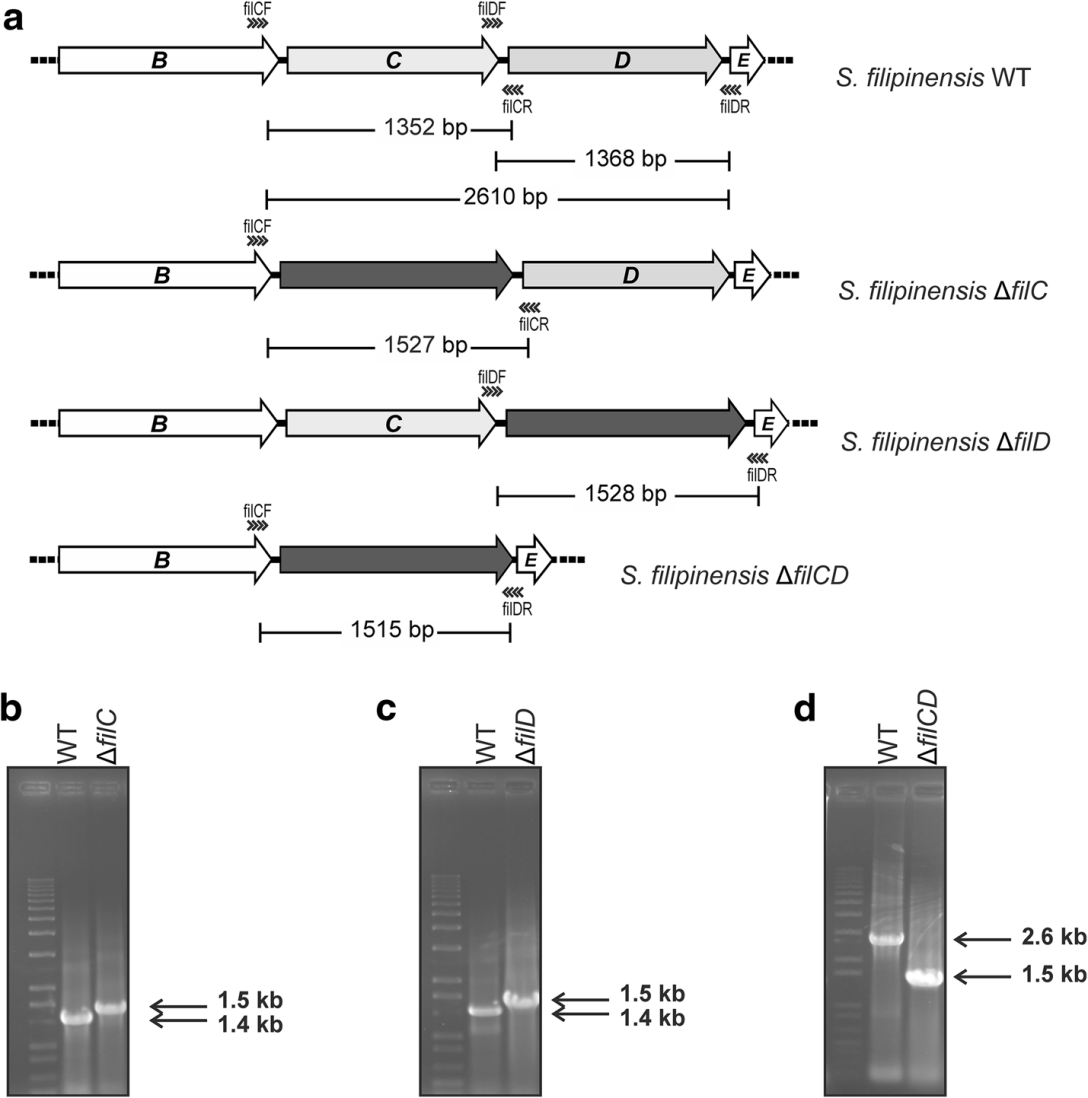
Construction of cytochrome P450 mutants. **a** Predicted PCR fragment amplification of the parental strain and the different mutants. The primers used in the assay are indicated with *arrowheads*. The *acc(3)IV*-*oriT* cassette is indicated in *black*. **b**–**d** PCR analysis of the wild type and the mutants.

In order to determine the function of *filC*, and *filD*, we inactivated them by using the REDIRECT gene replacement technology as indicated in “Methods”. Double-crossover mutants were screened by apramycin resistance. These were verified by PCR analysis (Fig. 3). Additionally, the double mutant *S. filipinensis ΔfilCD* was also constructed using the same methodology.

The new *S. filipinensis ΔfilC*, *ΔfilD*, and *ΔfilCD* strains had growth and morphological characteristics identical to those of *S. filipinensis* wild type when grown on solid or liquid media, suggesting that those genes had no role in bacterial growth or differentiation. The spore counts of all strains were similar after growth for 9 days at 30°C on TBO plates. The spores of all strains were serially diluted and plated on minimal medium to check their viability, finding no differences between them. All strains grew well in the liquid minimal medium, showing identical growth kinetics.

The fermentation broths produced by the new mutant strains, when grown in YEME medium, were extracted with ethyl acetate and analysed by thin layer chromatography for the presence of filipin III derivatives (see “Methods”). Figure 3 shows that the three mutant strains accumulated major compounds different to the filipin III produced by the parental strain, thus proving that the deleted genes were indeed filipin tailoring genes. To further characterize these compounds, we subjected extracts to high performance liquid chromatography (HPLC) analyses.

**Fig. 4:**
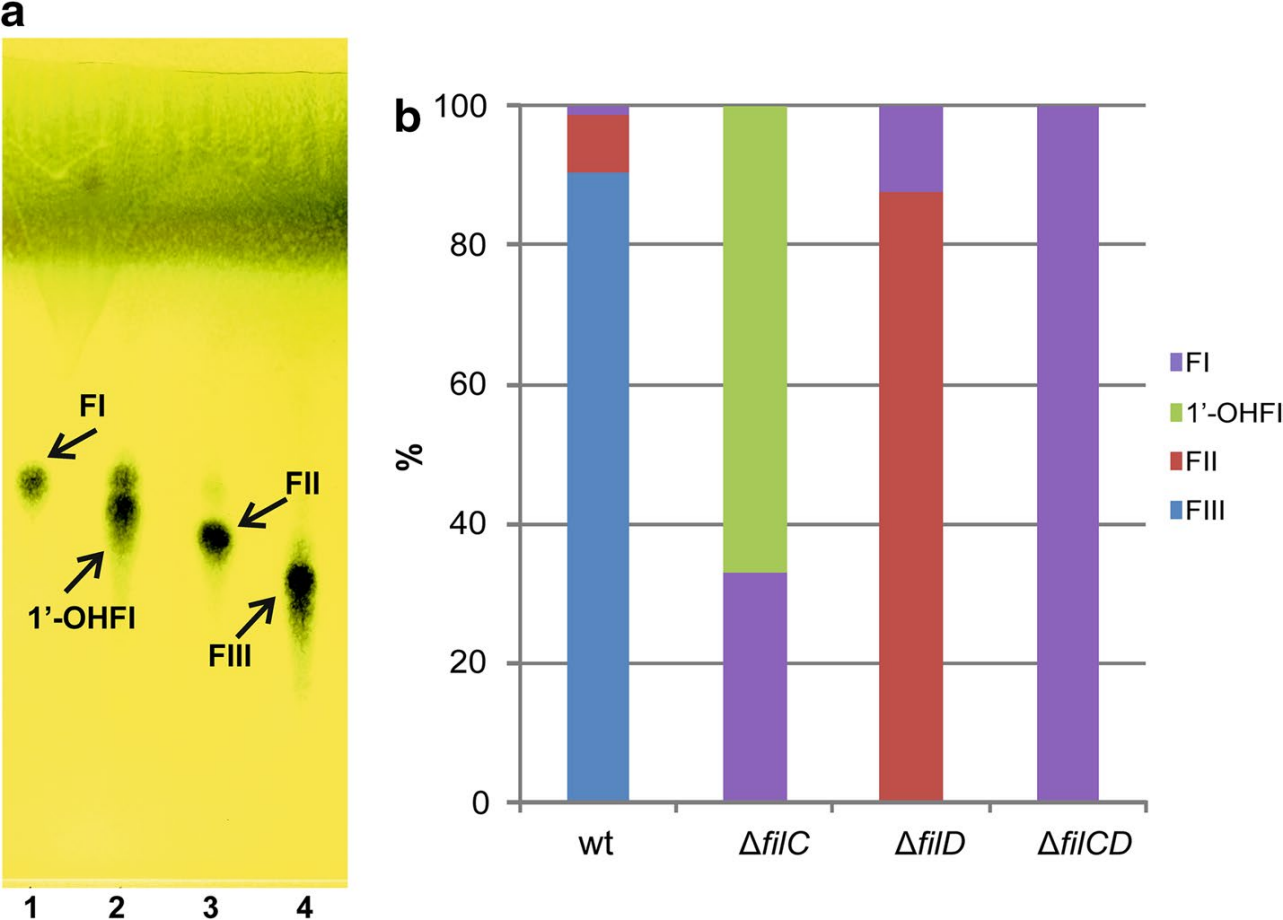
Filipin intermediates generated upon gene inactivation. **a** TLC analyses of 72 h culture broth extracts of *S. filipinensis* (*4*), *S. filipinensis ΔfilC* (*2*), *S. filipinensis ΔfilD* (*3*), and *S. filipinensis ΔfilCD* (*1*). **b** Relative proportions of the major filipin products accumulated by the different strains. FI, filipin I (compound Y); FII, filipin II (compound Z); FIII, filipin III; 1´-OHFI, 1´-hydroxyfillipin I (compound X).

After 72 h of growth, *S. filipinensis ΔfilC* mutant accumulated two major products, compounds X (ca. 67%) and Y (ca. 33%) (Fig. 4). Compound X was not observed in fermentation broths of the parental strain, whereas Y was produced at a very low proportion (ca. 1%). *S. filipinensis ΔfilD* also produced two major products, compounds Y (ca. 12%) and Z (88%) (Fig. 4), both present in fermentation broths of the parental strain albeit at very low proportion (1 and 6% respectively). In turn, the double mutant *S. filipinensis ΔfilCD* accumulated a single major product (100%) with the same retention time of compound Y (Fig. 4).

### Structural characterization of filipin III intermediates

Identification of compounds X, Y and Z was carried out by Mass Spectrometry (MS) followed by 1D and 2D Nuclear Magnetic Resonance (NMR) spectroscopy.

Mass Spectrometry analysis (Additional file 2: Figure S1) of compounds X, Y and Z yielded lower masses than filipin III (*m*/*z* 654.4), which were in agreement with the loss of one oxygen atom for compounds X (*m*/*z* 638.6) and Z (*m*/*z* 638.5), and with the loss of two oxygen atoms for compound Y (*m*/*z* 622.5).

^1^H and ^13^C-NMR data of compounds X, Y and Z were virtually identical to those of filipin III. The only differences were in the resonances of the C1′ and C26 positions (Table 1). HMBC and ^1^H,^1^H-COSY correlations were used to assign the C1′ (δ_H_, δ_C_) and C26 (δ_H_, δ_C_).

**Table 1:** MS and selection of ^1^H and ^13^C-NMR data of compounds X, Y, Z and filipin III

Compound	MS	^1^H and ^13^C-NMR position	
		26	1′
X (1′-hydroxyfilipin I)	[M+Na]^+^ 661.6	δ_H_: 2.24 and 2.29 δ_C_: 37.94	δ_H_: 3.72 δ_C_: 70.47
Y (filipin I)	[M+Na]^+^ 645.5	δ_H_: 2.25 and 2.35 δ_C_: 37.89	δ_H_: 1.44 and 1.68 δ_C_ : 29.47
Z (filipin II)	[M+Na]^+^ 661.5	δ_H_: 3.93 δ_C_: 72.11	δ_H_: 1.43 and 1.68 δ_C_: 29.60
Filipin III	[M+H]^+^ 655.4	δ_H_: 3.94 δ_C_: 73.00	δ_H_: 3.66 δ_C_: 69.50

Only the chemical shifts of the most relevant signals are listed. Positions are labelled according to their number in the polyketide backbone (Fig. 6).

HMBC spectrum of compound X showed correlations between the signal assignable to carbonyl group at 171.68 (C1) and the signals corresponding to H-1′ (δ_H_ 3.72) and H-3 (δ_H_ 4.00), proton chemical shifts according to hydroxylation at C1′ and C3. ^1^H,^1^H-COSY exhibited resonances of diastereotopic methylene protons H-2′ (δ_H_ 1.26, δ_H_ 1.31) with H-1′, which allowed to unambiguous assignment to CH-1′. The carbon chemical shifts of C-1′ (δ_C_ 70.47) were assigned by HSQC-HMQC spectrum. The absence of oxygenated function on C26 was stablished by HMBC correlations between CH_3_-28 (δ_H_ 1.17, δ_C_ 20.31) and CH_2_-26 (δ_H_ 2.24 and 2.29, δ_C_ 37.94). The complete assignments of NMR data are shown in Additional file 3: Table S2 (δ_H_, δ_C_, DEPT) and Additional file 4: Figure S2 (^1^H,^1^H-COSY and HMBC).

Similarly, were identified the compound Y as filipin I (Table 1). The resonances of C1′ (δ_H_ 1.44 and 1.68, δ_C_ 29.47) and C26 (δ_H_ 2.25 and 2.35, δ_C_ 37.89) were absent of hydroxyl group in these positions.

Finally, the compound Z was identified as filipin II by NMR data. The resonance of C1′ (δ_H_ 1.43 and 1.68, δ_C_ 29.60) was absent of hydroxyl function, while the position C26 (δ_H_ 3.93, δ_C_ 72.11) retained the oxygen atom.

### Gene complementation restores filipin III biosynthesis in the mutants

**Fig. 5:**
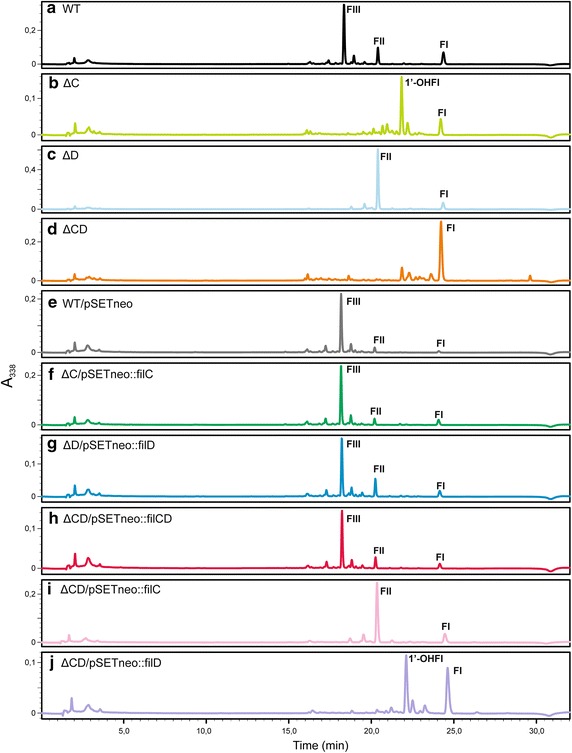
Analytical HPLC of products accumulated after gene deletions and after gene complementations. Comparison of HPLC analyses of ethyl acetate-extracted broths from *S. filipinensis* wild-type (**a**), and strains *S. filipinensis ΔfilC* (**b**), *S. filipinensis ΔfilD* (**c**), *S. filipinensis ΔfilCD* (**d**), *S. filipinensis* wt/pSETneo (**e**), *S. filipinensis ΔfilC*/pSETneo::filC (**f**), *S. filipinensis ΔfilD*/pSETneo::filD (**g**), *S. filipinensis ΔfilCD*/pSETneo::filCD (h), *S. filipinensis ΔfilCD*/pSETneo::filC (**i**), and *S. filipinensis ΔfilCD*/pSETneo::filD (**j**). Detection was carried out at *A*_338_. Abbreviations are as in Fig. 4.

To confirm that the gene deletions were directly responsible for the impairment on filipin III production and the accumulation of the intermediates described above, we complemented all mutants with the corresponding gene/s. For that purpose, we introduced one copy of *filC* into the genome of *S. filipinensis ΔfilC* using the integrative plasmid pSETneo::filC (see “Methods”). pSET152neo was also introduced into *S. filipinensis* wild type as control. Interestingly, introduction of the vector restored completely the ability of *S. filipinensis* Δ*filC* to produce filipin III (Fig. 5). Similarly, when we introduced one copy of *filD* into the genome of *S. filipinensis ΔfilD* using the integrative plasmid pSETneo::filD, or when we introduced single copies of *filC* and *filD* into the genome of *S. filipinensis ΔfilCD* using the integrative plasmid pSETneo::filCD (see “Methods”) a total restoration of filipin III production was observed (Fig. 5).

Introduction of an extra copy of *filD* in *S. filipinensis ΔfilC*, or an extra copy of *filC* in *S. filipinensis ΔfilD*, did not modify their HPLC profile (not shown), thus indicating that the P450 monooxygenases they encode show strict regiospecificity, as has been demonstrated for their counterparts from *S. avermitilis* [14].

Strikingly, introduction of a copy of *filC* into the double mutant resulted in the production of filipin II, as the major product, by the recombinant strain, whereas introduction of *filD* into the same strain resulted in the accumulation of similar amounts of both filipin I and 1′-hydroxyfilipin I (Fig. 5). These results are in concordance with those observed upon inactivation of the individual genes, thus corroborating results above.

### There are two alternative routes for filipin III biosynthesis

**Fig. 6:**
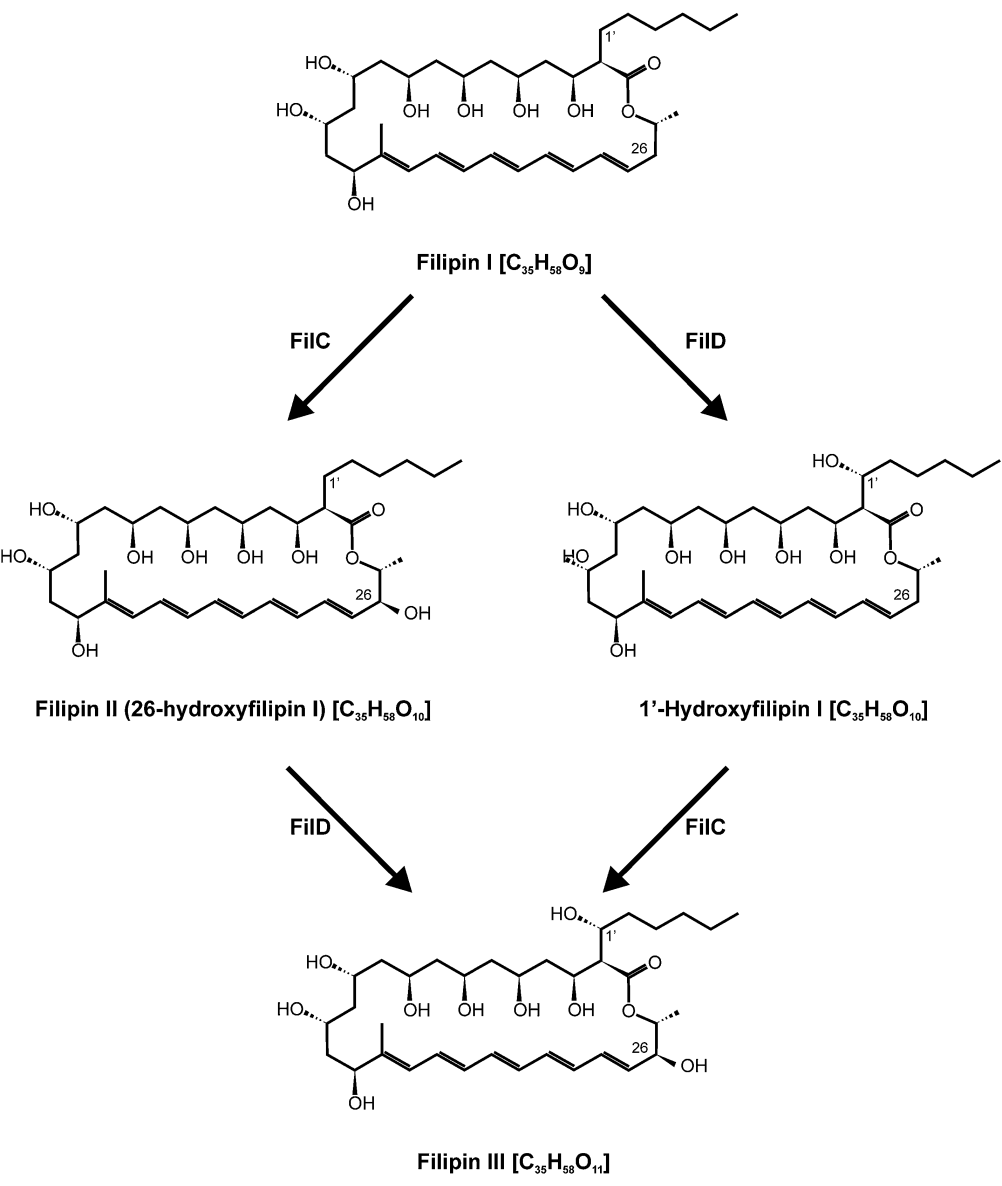
Two alternative routes on filipin III biosynthesis in *S. filipinensis.*

The production of filipin I as a sole product by the double mutant indicates that filipin I is the aglycone resulting from the polyketide synthases assembly line after cyclization. This is in agreement with the model proposed by Ikeda et al. [2] for *S. avermitilis*. In their model, filipin I is first hydroxylated at C26 to yield filipin II, and then filipin II is further hydroxylated at C1′ to yield filipin III. The major production of filipin II (ca. 88%) by the *S. filipinensis ΔfilD* mutant (Fig. 4) indicates that filipin I is the substrate of FilC which hydroxylates the polyene at C26 (Fig. 6). This result somewhat demonstrates in vivo what had already been shown in vitro with recombinant CYP105P1 (PteC) that converts 50% of filipin I to filipin II [14]. Interestingly, *S. filipinensis ΔfilC* mutant produced filipin I as expected, but also a large proportion (about 67%) of 1′-hydroxyfilipin I (Fig. 4). This indicates that in vivo FilD is able to hydroxylate filipin I at C1′, as has been shown in vitro by recombinant CYP105D6 (PteD) [14]. These results were corroborated by gene complementation of the double mutant with *filC*, thus validating that FilC catalyzes the conversion of filipin I to filipin II, and with *filD*, confirming that FilD can convert filipin I to 1′-hydroxyfilipin I. Remarkably, gene complementation results also demonstrate that both filipin II and 1′-hydroxyfilipin I are converted to filipin III, thus indicating that there are two alternative routes for filipin III formation (Fig. 6). In one of the alternatives of the route, filipin I is converted to filipin II by FilC, and the latter is then converted to filipin III by FilD, as in the model proposed by Ikeda et al. [2]. In the second option, filipin I is first converted to 1′-hydroxyfilipin I by FilD, which is subsequently converted to filipin III by FilC (Fig. 6).

### Yield and bioactivity of filipin III intermediates

**Fig. 7:**
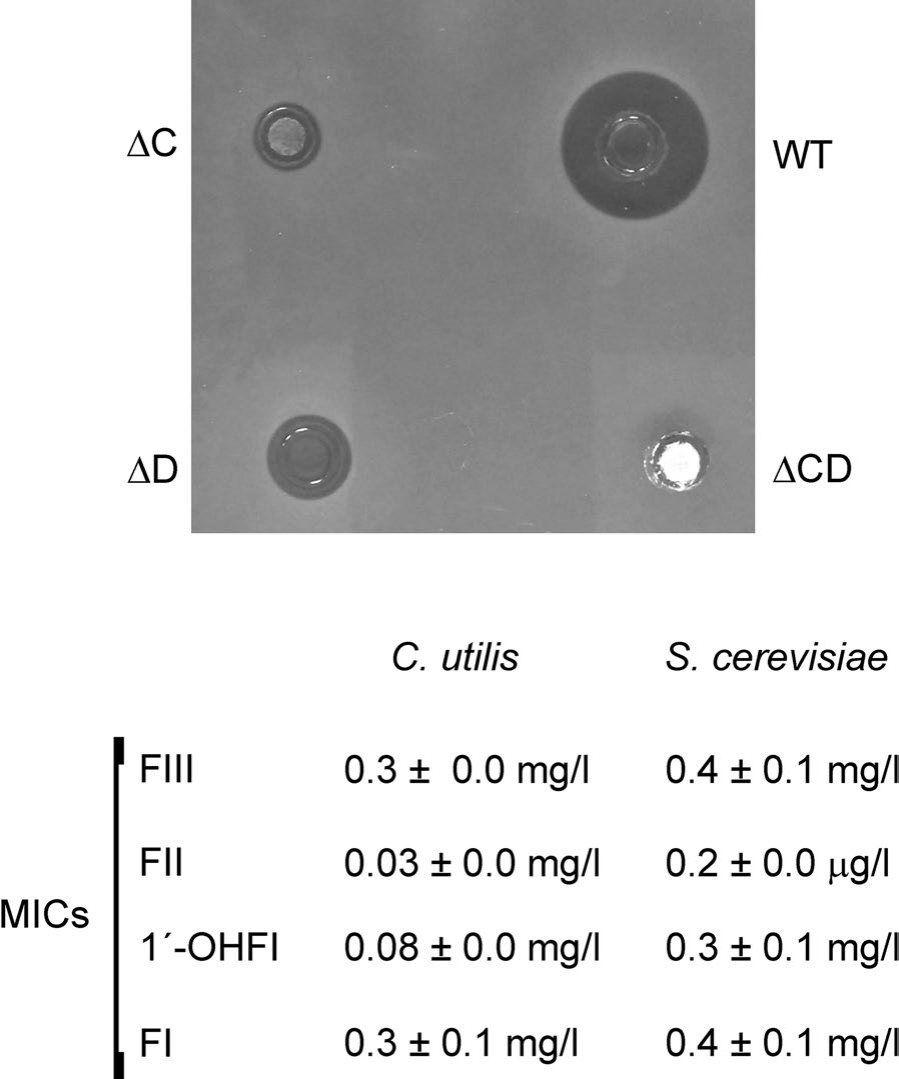
Bioassay and MICs of filipin III and its intermediates. Bioassays were performed with ethyl acetate-extracted culture broths from early stationary phase-grown cells. *C. utilis* was used as test organism. MICs of the purified filipin derivatives for *C. utilis* and *S. cerevisiae* were measured by broth microdilution assay (see “Methods”). Abbreviations are as in Fig. 4.

Comparison of the amount of major products produced by the different strains indicated that the yields of polyenes in YEME medium without sucrose were very similar for the four strains, in the range of 50 μg of product per mg of dry cells (around 160 mg/l). In order to characterise the biological activity of the products, we initially compared bioassays performed with ethyl acetate-extracted culture broths from early stationary phase-grown cells of the mutants with those of the parent strain. In the case of *S. filipinensis ΔfilD*, we found a consistent halo of growth inhibition of the *C. utilis* cells used as test organism (Fig. 7), indicating that filipin II retains antibiotic activity. This halo was smaller than that of filipin III, thus either filipin II has lower antibiotic activity than filipin III or the compound displays a smaller diffusion rate in agar than the latter. In the case of *S. filipinensis ΔfilC*, we also found an halo of inhibition of *Candida* growth although smaller than that of filipin II, indicating that 1′-hydroxyfilipin I also retains antibiotic activity (Fig. 7). In turn, *S. filipinensisΔfilCD* yielded almost unappreciable halos under the conditions used. Similar results were observed when we used *S. cerevisiae* as test organism.

Filipin III intermediates were then purified, and minimal inhibitory concentrations were measured by broth microdilution assay, finding MICs for *C. utilis* of 0.3 mg/l for filipin III and filipin I, 0.08 mg/l for 1′-hydroxyfilipin I, and 0.03 mg/l for filipin II (Fig. 7). *S. cerevisiae* turned out to be less sensitive, but the bioactivity of the compounds followed the same pattern (Fig. 7). The lower MICs of both filipin II and 1′-hydroxyfilipin I when compared with that of filipin III indicate that these new compounds show remarkably improved bioactivity against *C. utilis*, and given their bioassay results also suggest a lower diffusion rate than filipin III.

## Discussion

The organization of the filipin tailoring genes identified in *S. filipinensis* turned out to be identical to that of *S. avermitilis*, except for the presence of a PadR-like encoding gene in one of the fringes of the *S. filipinensis* cluster that is absent in *S. avermitilis*. Given that this gene constitutes the main difference between both clusters, one could speculate that the enormous difference in production between both strains could be attributable to such gene. PadR belongs to a family of transcriptional regulators (Pfam PF03551), usually repressors that regulate diverse processes [24, 25, 27], although activators have also been described [26], and among those processes could be the production of the antifungal filipin. In fact, gene disruption experiments indicated that FilI is actually involved in filipin biosynthesis (Fig. 2). However, filipin production was boosted in the knocked-out mutant when compared with the parental strain, thus indicating that FilI acts as a negative regulator of the biosynthesis. How a negative regulator could improve antifungal production is difficult to interpret. FilI may act either directly as a negative regulator on the promoters of given *fil* genes, or it may activate or repress a hierarchical regulator controlling *fil* genes expression, but none of these possibilities can explain the higher filipin III production of *S. filipinensis*, hence a different explanation must be sought. Future experimental analyses will hopefully provide an answer to this question. Noteworthy, while writing this manuscript several *S. durhamensis* sequences showing homology to *fil* genes have been deposited in the databases in the context of a sequencing project (GenBank: NZ_JNXR00000000.1). Those genes show the same organization as the *fil* genes including the *padR*-like regulatory gene neighboring a discrete thioesterase gene.

Besides the presence of an additional regulatory gene, the difference in polyene yield between both strains could also be explained by the changes observed in the intergenic regions that could also have effects on the gene transcription given that these regions may include promoter activities. In fact, in *S. avermitilis* there are promoters immediately upstream *pteD* and *pteH* [9], while we have not detected them in *S. filipinensis*. In turn, in *S. filipinensis*, *filF* and *filG* belong to different transcriptional units, whereas *pteG* can either be co-transcribed with *pteF* or use its own promoter in *S. avermitilis* [9]. A similar situation has been described for the pimaricin producers *S. natalensis* and *S. chattanoogensis* that show different transcriptional organization due to changes in intergenic regions [28].

*Streptomyces filipinensis* mutants in P450 monooxygenase encoding genes *filC* and *filD*, and in both (*filCD*), were obtained by PCR-targeting. The strains so generated produced filipin III derivatives as shunt metabolites, thus establishing the link of these genes with filipin III biosynthesis. The double mutant accumulated filipin I as a sole product, whereas complementation with single copies of both *filC* and *filD* restored filipin III biosynthesis, thus demonstrating that filipin I is the direct product of PKS assembly and cyclization. The production of filipin I and 1′-hydroxyfilipin I (26-deoxyfilipin III), as major products, by *S. filipinensis* Δ*filC*, demonstrates that FilD is in fact the enzyme responsible for the introduction of an hydroxyl function at C1′, whereas the production of filipin I and filipin II by *S. filipinensis* Δ*filD*, indicates that FilC is in charge of the introduction of the hydroxyl group at C26. Gene complementation of the mutants with a single copy of the corresponding gene restored wild-type phenotype, whereas cross-complementation was unsuccessful, indicating that both enzymes show strict regiospecificity. These results are in agreement with the in vitro results obtained with *S. avermitilis* recombinant PteC and PteD which catalyse filipin hydroxylation at C26 and C1′ respectively [14].

Notably, our results also indicate that there are two alternative routes for filipin III formation (Fig. 6). In one of the alternatives, filipin I is converted to filipin II by FilC, and the latter is then converted to filipin III by FilD, as in the model proposed by Ikeda et al. [2]. In the second option, filipin I is first converted to 1′-hydroxyfilipin I by FilD, which is subsequently converted to filipin III by FilC. Which one is the preferential option remains to be elucidated, although according to the complementation results of the double mutant *S. filipinensis ΔfilCD* with the individual genes (Fig. 5), FilC seems to hydroxylate Filipin I at a higher rate than FilD. This is in agreement with the in vitro results observed with the *S. avermitilis* enzymes PteC and PteD [14]. Besides, 1′-hydroxyfilipin I was never observed in the wild-type strain fermentation broths, thus suggesting that FilC converts all of this intermediate to filipin III in vivo.

Identification of filipin III intermediates has been carried out by MS and various NMR techniques, and this report constitutes the first description of the complete nuclear magnetic resonance assignments for 1′-hydroxyfilipin I. Strikingly, all filipin derivatives were accumulated at high yield by the mutants, which will allow the massive production of filipin III intermediates that will prove valuable as precursors for the synthetic generation of novel derivatives.

All the derivatives obtained (filipin I, filipin II, and 1′-hydroxyfilipin I) were biologically active. While filipin I showed a similar MIC to filipin III (0.3 or 0.4 mg/l depending of the test organism), the antifungal activity of 1′-hydroxyfilipin I was 3.75 or 1.33 times higher (for *C. utilis* and *S. cerevisiae* respectively) and that of filipin II the highest, being tenfold or twofold than that of filipin III (for *C. utilis* and *S. cerevisiae* respectively). The contrast between MICs of filipin derivatives with the sizes of the growth inhibition zones in bioassays could be attributed to the different diffusion rates of the intermediates in agar. Diffusion rate of antibiotics is a fundamental parameter of agar diffusion assays [29]. Thus, compounds having a good diffusion coefficient and low antimicrobial activity may penetrate the agar medium even in small amounts, and the reverse also holds true. Filipin III has one extra hydroxyl group when compared with filipin II or 1′-hydroxyfilipin I, therefore it is more polar, and its diffusion rate is higher than those of its precursors. This lack of correlation between MIC measurement and agar diffusion tests has also been reported in studies with substituted salicylaldehydes, where compounds with no activity in agar diffusion tests had potent activity in MIC tests [30], and therefore must be taken into consideration for antimicrobial activity screening. The higher antifungal activity of intermediates of the pathway was unexpected given that, normally, the final product is the compound with greater bioactivity [31, 32]. But considering that filipin III shows a similar affinity for cholesterol and ergosterol [33], it is likely that both filipin II and 1′-hydroxyfilipin I show an improved affinity for ergosterol than filipin III. Future analysis of other parameters important for the applicability of these designer polyenes such as host-range, toxicity, and acid or alkali stability will establish the pharmaceutical importance of the findings presented here.

## Conclusions

Genetic manipulations of polyene biosynthetic pathways have proven useful for the discovery of products with improved properties. Here, we describe the late biosynthetic steps for the biosynthesis of the pentaene macrolide filipin III, finding that two alternative routes lead to its formation. In one of the routes, filipin I is converted to filipin III via filipin II, whereas in the other, the intermediate is 1′-hydroxyfilipin I, an unnatural compound. All intermediates were produced at high yield, and showed antifungal activity, being 1′-hydroxyfilipin and especially filipin II remarkably more active than filipin III. These results open new possibilities for the generation of biologically active filipin derivatives at high yield and with improved properties.

## Methods

### Bacterial strains and cultivation

*Streptomyces filipinensis* DSM 40112 was used as the source of DNA in the construction of the genomic library, and was routinely grown in YEME medium [34] without sucrose. Sporulation was achieved as described elsewhere [35]. *Escherichia coli* strain DH5α was used as a host for DNA manipulation. *E. coli* strain XL1-Blue MR was used for obtaining SuperCos 1 cosmid (Stratagene) recombinant derivatives. *E. coli* BW25113 [pIJ790] was used for gene replacement experiments. *E. coli* ET12567 [pUZ8002] was used as donor in intergeneric conjugations. *C. utilis* (syn. *Pichia jadinii*) CECT 1061 and *S. cerevisiae* CECT 1942 were used for bioassay experiments. Minimum inhibitory concentrations (MICs) were determined by the broth microdilution technique following the EUCAST methodology by diluting filipin III derivatives in RPMI 1640 with glutamine and 0.2% glucose but without sodium bicarbonate (Sigma) buffered with 0.164 M MOPS pH 7.0 to concentrations of 40 μg/ml of which 100 μl was added to the first row of a 96-well suspension culture plate. This was followed by a 1:1 dilution series in medium. The plates were spotted with 2,000 viable *C. utilis* or *S. cerevisiae* cells. The MIC value was determined to be the lowest concentration of antibiotic, which inhibited the growth of the yeast strain and could be determined by eye on the 96-well plate after an incubation of 24 h at 30°C. Commercial filipin III (Sigma) was used as control.

### Plasmids and DNA manipulation procedures

Standard genetic techniques with *E. coli* and in vitro DNA manipulations were as described by Sambrook and Russell [36]. Recombinant DNA techniques in *Streptomyces* species and isolation of *Streptomyces* total DNA were performed as previously described [34]. For construction of the genomic library, *S. filipinensis* genomic DNA was partially digested with *Sau*3AI and fragments in the 35 ± 40 kb size range were cloned into SuperCos 1 digested with *Bam*HI and *Xba*I. The ligation mixture was packaged with Gigapack III XL (Stratagene) and used to transfect *E. coli* XL-1 Blue MR. The library was screened using probes obtained by PCR amplification of *S. filipinensis* chromosomal DNA by using oligonucleotides derived from conserved stretches of the filipin PAS-LuxR regulator *pteF* from *S. avermitilis* [9]. The oligonucleotide pairs used were 5′-CGGCTCGTCCGAGGACATATGC-3′ and 5′-AATCACGCTGTGGCTCCTGAGCTCGGG-3′. A positive clone (Cos 8G9) containing a *pteF* homologous gene was used as the starting point for chromosome walking. Southern hybridization was carried out with probes labelled with digoxigenin by using the DIG DNA labelling kit (Roche Biochemicals). Intergeneric conjugation between *E. coli* ET12567 [pUZ8002] and *S. filipinensis* was performed as described [37]. pUC19 (New England Biolabs) was used as the routine cloning vector, pSETneo (Am^R^, Kan^R^, pUC18 replicon, ΦC31 *attP*; [38]), was used for intergeneric conjugation. Polymerase chain reactions were carried out using Phusion DNA polymerase as described by the enzyme supplier (Finnzymes). DNA sequencing was accomplished by the dideoxynucleotide chain-termination method using the Perkin Elmer Amplitaq Gold Big Dye-terminator sequencing system with an Applied Biosystems ABI 3130 DNA genetic analyzer (Foster City, CA, USA).

### RNA isolation and reverse transcriptase-PCR experiments

RNA was isolated as described [9]. Transcription was studied by using the SuperScript™ One-Step reverse transcriptase-PCR (RT-PCR) system with Platinum^®^ Taq DNA polymerase (Invitrogen), using 200 ng of total RNA as template. Conditions were as follows: first strand complementary DNA (cDNA) synthesis, 50°C for 40 min followed by heating at 94°C for 2 min; amplification, 40 cycles of 98°C for 15 s, 59–68°C (depending of the set of primers used) for 30 s, and 72°C for 1 min. Primers (18–28 mers; Additional file 1: Table S1) were designed to cover intergenic regions, generating PCR products of approximately 100–650 bp. Negative controls were carried out with each set of primers and Platinum^®^ Taq DNA polymerase in order to confirm the absence of contaminating DNA in the RNA preparations. The identity of each amplified product was corroborated by direct sequencing of the PCR product.

### Construction of Δ*filC*, Δ*filD*, Δ*filCD*, and Δ*filI* mutants

Deletion of *filC* from *S. filipinensis* chromosome was made by replacing the wild-type gene with a cassette containing an apramycin selective marker using a PCR based system [39]. The plasmid pIJ773 containing the apramycin resistance gene (*aac(3)IV*) and the *oriT* replication origin was used as a template. The mutant was constructed using the oligonucleotides (CDF) 5′-*gtacgacccccccacccacaagctccaaggagagccatg*ATTCCGGGGATCCGTCGACC-3′ and (CR) 5′-gggcggtgcgggcgtgcgtcgagatgtgaggccgggtcaTGTAGGCTGGAGCTGCTTC-3′ as the forward and reverse primers respectively (the sequence identical to the DNA segment upstream from the start codon of *filC* is in lower case italics and the sequence identical to the segment downstream from the stop codon of *filC* is underlined and in lower case). These two long PCR primers (59 and 58 nt) were designed to produce a deletion of *filC* just after its start codon leaving only its stop codon behind. The 3′ sequence of each primer matches the right or left end of the disruption cassette (the sequence is shown uppercase in both primers). The extended resistance cassette was amplified by PCR and *E. coli* BW25113/pIJ790 bearing cosmid 8C8 was electro-transformed with this cassette. The isolated mutant cosmid was introduced into non-methylating *E. coli* ET12567 containing the RP4 derivative pUZ8002. The mutant cosmid was then transferred to *S. filipinensis* by intergeneric conjugation. Double cross-over exconjugants were screened for their apramycin resistance followed by confirmation by PCR.

Mutant Δ*filD* was constructed following the same strategy, using the oligonucleotides (DF) 5′-*gcacgcccgcaccgccccttgctcgaaaggcaccacatg*TGTAGGCTGGAGCTGCTTC-3′ and (CDR) 5′-ccgccaccctttccttccgttccgtccggccgggccgtcaATTCCGGGGATCCGTCGACC-3′ as the forward and reverse primers respectively, and the same cosmid template. Similarly, mutant Δ*filCD* was constructed using oligonucleotides CDF and CDR. Mutant Δ*filI* was constructed using oligonucleotides (IF) 5′-ttgctatgcaacgagttgcatagcaggatcgacggcatgATTCCGGGGATCCGTCGACC-3′ and (IR) 5′-ctggaccggcgggacctgcgggcggtacggcggcgatcaTGTAGGCTGGAGCTGCTTC-3′, and cosmid 8G9 as template.

In all cases, mutants were verified by PCR analysis (Figs. 2, 3).

### Construction of plasmids for gene complementation

In order to complement *filC* replacement mutant, a 1,352 bp DNA fragment containing the entire *filC* gene plus its upstream intergenic region was amplified by PCR with primers filCF (5′-GGAATTCGCTCGCCGCCGCCTGAC-3′) and filCR (5′-GGAATTCGGCGGATGTCGGTGTCGGTC-3′) using *S. filipinensis* chromosomal DNA as template. The PCR product was cloned into an *Eco*RI-cut pSETneo, and the construction with the gene in the same orientation of the *neo* gene was selected to yield pSETneo::filC. This would permit gene expression driven from the promoter of the *neo* gene in case the cloned DNA fragment lacked promoter activity.

Similarly, for *S. filipinensis* Δ*filD* gene complementation, a 1,368 bp DNA fragment containing the entire *filD* gene plus its upstream intergenic region was amplified by PCR with primers filDF (5′-GGAATTCGAGCTGCCCGTCACCTGG-3′) and filDR (5′-GGAATTCGCGGTCGATGGTGATGCG-3′). The PCR product was cloned into an *Eco*RI-cut pSETneo to yield pSETneo::filD after selection of gene orientation.

Similarly, for the complementation of the double mutant, a 2.6 kb DNA fragment containing *filC* and *filD* was amplified by PCR with primers filCF and filDR. The PCR product was then cloned into the *Eco*RI site of pSETneo to yield pSETneo::filCD.

### Assay of filipin production

To determine filipins production and metabolite purification, one volume of culture was extracted with two volumes of ethyl acetate, and the organic phase was sequentially treated with saturated NaCl solution and Na_2_SO_4_, vacuum dried, and resuspended in pure methanol. For routine determination of metabolite yield, cultures were extracted with one volume of methanol, and diluted when needed. Solutions of pure filipin III (Sigma) were used as control. Quantitative determination of filipins was assessed by reverse phase HPLC using a Waters 600 unit coupled to a diode array ultraviolet detector set at 338 nm equipped with a Mediterranean Sea C18 column (4.6 × 150 mm, particle size 3 µm) (Teknokroma). Elution was performed with a gradient (0.8 ml/min) of methanol-0.1% formic acid according to the following program (50:50 v/v 0–3 min, up to 90:10 v/v 3–12 min, 90:10 12–20 min, up to 100:0 v/v 20–21 min, 100:0 v/v 21–23 min, down to 0:100 v/v 23–24 min, 0:100 v/v 24–26 min, up to 50:50 v/v 26–27 min, 50:50 v/v 27–32 min). Retention time for Filipin III was 18.2 min. Thin layer chromatography was performed on silica 60 F254 plates (Merck), and elution was carried out with dichloromethane:methanol (4:1) (v/v). Plates were developed by spraying with phosphomolybdic acid:ethanol (1:9) (v/v) and heating.

### Structural elucidation of 1′-hydroxyfilipin I

Fermentation broths of *S. filipinensis ΔfilC* were harvested by centrifugation after 3 days of growth at 250 rpm and 30°C, and the supernatant treated as indicated above. For MS analyses, the dry residue was dissolved in methanol, whereas for nuclear magnetic resonance (NMR) determination, the dry residue was dissolved in deuterated dimethyl sulfoxide.

Mass spectra experiments were taken on an Ultrafex III MALDI TOF/TOF apparatus (Bruker) by using dithranol + NaI as the matrix and FAB-MS spectrum was recorded on a VG Autospectrum (Waters) instrument using *m*-nitrobenzyl alcohol (*m*-NBA) as the matrix and Caesium (Cs^+^) as ion bombardment at 35 kV. A filipin III solution (5 mg/ml methanol) was used for tuning.

The structure was elucidated on the basis of the 1D-NMR: ^1^H-NMR, ^13^C-NMR and DEPT (Distortionless Enhancement by Polarization Transfer), and 2D-NMR: COSY (H,H-Correlation Spectroscopy), HSQC (Heteronuclear Single Quantum Coherence), HMQC (Heteronuclear Multiple Quantum Correlation), and HMBC (Heteronuclear Multiple Bond Correlation) experiments, and by comparison with the spectrum of filipin III [40]. NMR spectra were recorded in DMSO-*d*_6_ at room temperature using a Bruker WM500 spectrometer [500 MHz (^1^H-NMR) and 125 MHz (^13^C-NMR)]. The pulse programmes of the two-dimensional experiments were taken from the Bruker software library, and the parameters were as follows: 500/125 MHz gradient-selected HMQC spectra: relaxation delay *D*1 = 1.5 s; 500/125 MHz gradient-selected HMBC spectra: relaxation delay *D*1 = 1.5 s, evolution delay *D*2 = 3.33 ms; delay for evolution of long-range coupling *D*6 = 60 ms. 500 MHz gradient-selected ^1^H,^1^H COSY spectra: relaxation delay D1 = 1.5 s; 90º pulse for ^1^H.

### Accession number

The sequence has been deposited in the GenBank database under the accession number KP769541.

## Additional files

Additional file 1: Table S1. Primers used in RT-PCR experiments. Additional file 2: Figure S1. MS identification of filipin intermediates. A) MALDI TOF/TOF spectrum showing the molecular ion MNa+ (645.5) of compound Y (filipin I). B) MALDI TOF/TOF spectrum of compound Z (filipin II) (MNa+ = 661.5). C) FAB spectrum of compound X (1´-hydroxyfilipin I) showing the molecular ion MNa + (661.6) of the complete molecule. Additional file 3: Table S2.
^1^H and ^13^C- NMR chemical shifts assignments for 1´-hydroxyfilipin I and Filipin III. Additional file 4: Figure S2.
^1^H,^1^H-COSY and HMBC correlations of 1´-hydroxyfilipin I.
