# Supplementary material for: Functional analysis of filipin tailoring genes from Streptomyces filipinensis reveals alternative routes in filipin III biosynthesis and yields bioactive derivatives
Source: Microb Cell Fact. 2015 Aug 7;14:114. doi: 10.1186/s12934-015-0307-4 (PMC4527110; doi:10.1186/s12934-015-0307-4)
Supplement: Additional file 1: Table S1. — Primers used in RT-PCR experiments. [file 12934_2015_307_MOESM1_ESM.docx]

| **Primer** | **Sequence (5’ to 3’)** | **Intergenic region** |
| --- | --- | --- |
| RT11-F-Sf-InterA5-B  Seq24-R | GCGGCTGTGGACTCCTGGC  GCCCAGCGAGGACCACACG | *filA5-filB* |
| Compl-Sf-C-Fw  Seq10-R | GGAATTCGCTCGCCGCCGCCTGAC  GATGCGCTGGATGTTCGGC | *filB-filC* |
| Compl-D-Fw  Compl-Sf-C-Rv | GGAATTCGAGCTGCCCGTCACCTGG  GGAATTCGGCGGATGTCGGTGTCGGTC | *filC-filD* |
| Seq13-F  Compl-D-Rv | CGCACGCCAGTTGGAGGAGG  GGAATTCGCGGTCGATGGTGATGCG | *filD-filE* |
| compl-Sf-F-Fw  Seq1-R | GGAATTCGAGGTGTGGGCATGGTGGTCC  GGCTGCTGCACGCTCGGG | *filR-filF* |
| Seq3-F  Seq4-R | GGACGACTTCGTGAAGTGAGCGG  CGAACGTGCCGCCGGAACC | *filF-filG* |
| Seq7-Sf-F  Seq8-R | CTCATCCCCGGCAGCATCG  GTCGTCGTCGCGCAGGTGG | *filG-filH* |
| Seq9-F  RT-23-R-padR | GGCGACGACGACCCGAAGG  GTGCAGCCAGGAGGACAGCG | *filH-filI* |

**Table S1. Primers used in RT-PCR experiments.**
