# Supplementary figures and images for: Functional analysis of filipin tailoring genes from Streptomyces filipinensis reveals alternative routes in filipin III biosynthesis and yields bioactive derivatives
Source: Microb Cell Fact. 2015 Aug 7;14:114. doi: 10.1186/s12934-015-0307-4 (PMC4527110; doi:10.1186/s12934-015-0307-4)

**A**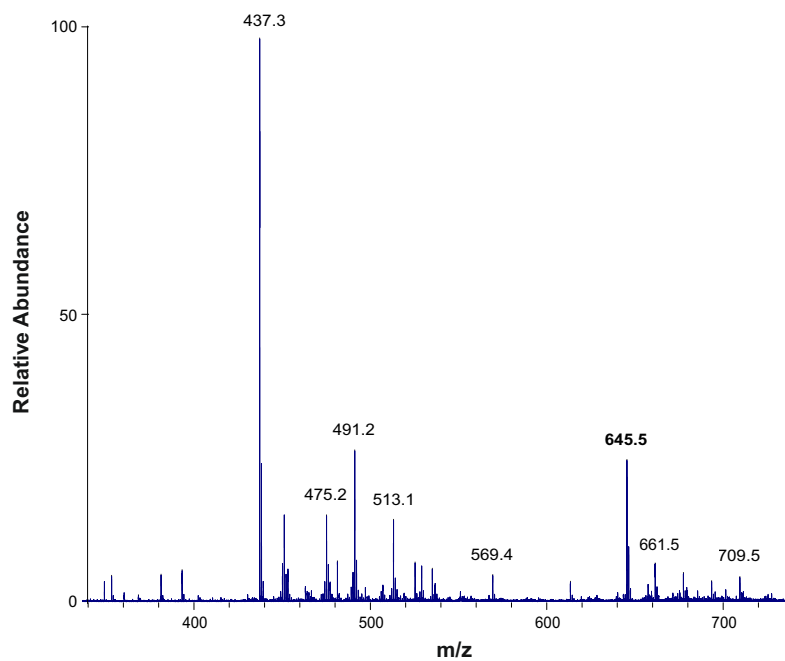**B**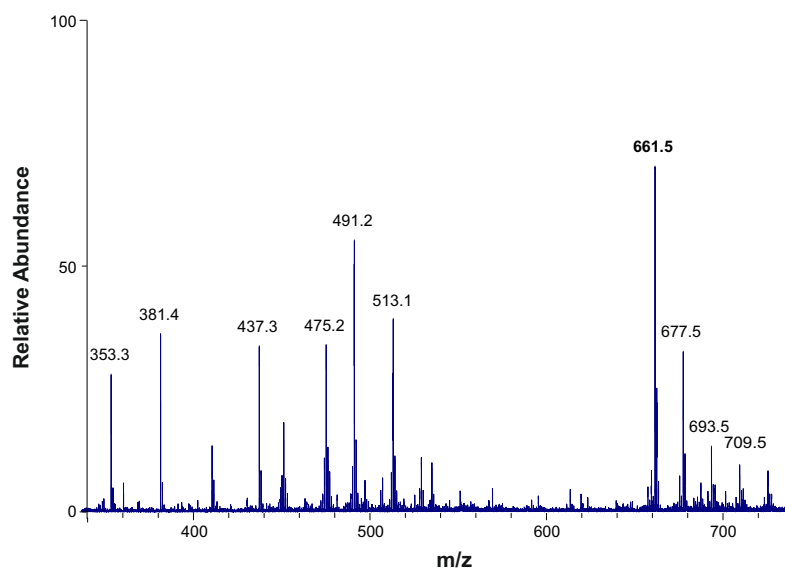**C**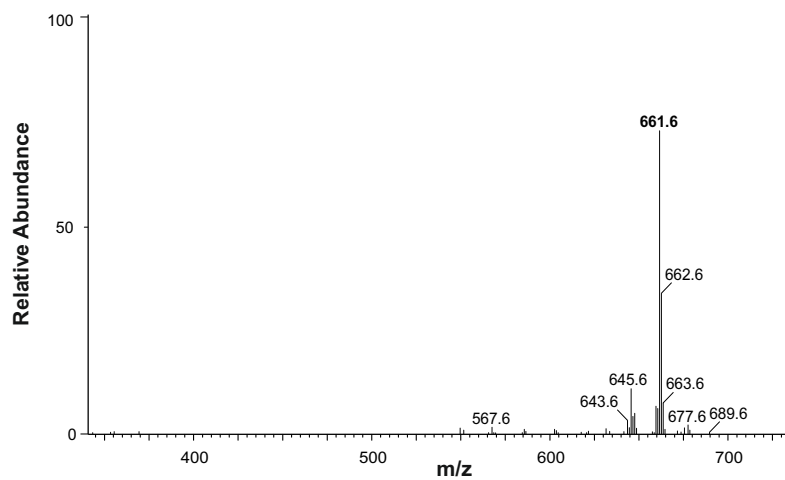

Supplement: Additional file 2: Figure S1. — MS identification of filipin intermediates. A) MALDI TOF/TOF spectrum showing the molecular ion MNa+ (645.5) of compound Y (filipin I). B) MALDI TOF/TOF spectrum of compound Z (filipin II) (MNa+ = 661.5). C) FAB spectrum of compound X (1´-hydroxyfilipin I) showing the molecular ion MNa + (661.6) of the complete molecule. [file 12934_2015_307_MOESM2_ESM.pdf]
