# Supplementary material for: Functional analysis of filipin tailoring genes from Streptomyces filipinensis reveals alternative routes in filipin III biosynthesis and yields bioactive derivatives
Source: Microb Cell Fact. 2015 Aug 7;14:114. doi: 10.1186/s12934-015-0307-4 (PMC4527110; doi:10.1186/s12934-015-0307-4)
Supplement: Additional file 3: Table S2. — 1H and 13C- NMR chemical shifts assignments for 1´-hydroxyfilipin I and Filipin III. [file 12934_2015_307_MOESM3_ESM.doc]

**Table S2. ^1^H and ^13^C- NMR chemical shifts assignments for 1´-hydroxyfilipin I and Filipin III.**

| **1´-hydroxyfilipin I** | | |  | **Filipin III** [40] | |
| --- | --- | --- | --- | --- | --- |
|  |
| **Nº** | **δC** (DEPT) | **δH** |  | **δC** (DEPT) | **δH** |
| 1 | 171.68 (C) |  |  |  |  |
| 2 | 58.88 (CH) | 2.43 (t, J= 8.05 Hz) |  | 58.1 (CH) | 2.44 |
| 3 | 71.00 (CH) | 4.00 (m) |  | 69.9 (CH) | 3.98 |
| 4 | 41.02 (CH_2_) | 1.36 (m) |  | 40.0 (CH_2_) | 1.36 (m) |
| 5 | 70.74 (CH) | 3.88 (m) |  | 69.4 (CH) | 3.87 |
| 6 | 44.06 (CH_2_) | 1.31 (m) |  | 43.4 (CH_2_) | 1.30 (m) |
| 7 | 70.67 (CH) | 3.86 (m) |  | 69.4 (CH) | 3.85 |
| 8 | 44.48 (CH_2_) | A: 1.38 (m)  B: 1.30 (m) |  | 43.4 (CH_2_) | A: 1.38  B: 1.30 |
| 9 | 71.69 (CH) | 3.85 (m) |  | 69.9 (CH) | 3.84 |
| 10 | 43.06 (CH_2_) | A: 1.37 (m)  B: 1.23 (m) |  | 42.3 (CH_2_) | A: 1.36  B: 1.26 |
| 11 | 69.39 (CH) | 3.80 (m) |  | 68.3 (CH) | 3.77 |
| 12 | 44.59 (CH_2_) | A: 1.60 (m)  B: 1.30 (m) |  | 44.3 (CH_2_) | A: 1.58  B: 1.28 |
| 13 | 65.66 (CH) | 3.07 (t br, J= 10.5 Hz) |  | 64.7 (CH) | 3.04 |
| 14 | 42.82 (CH_2_) | A: 1.72 (m)  B: 1.54 (m) |  | 42.4 (CH_2_) | A: 1.68  B: 1.49 |
| 15 | 73.80 (CH) | 3.97 (m) |  | 71.0 (CH) | 3.94 |
| 16 | 140.67 (C) |  |  |  |  |
| 17 | 126.37 (CH) | 5.92 (d, J= 11.2 Hz) |  | 125.4 (CH) | 5.91 |
| 18 | 128.43 (CH) | 6.45 (dd, J= 14.5 & 11.2 Hz) |  | 127.7 (CH) | 6.44 |
| 19 | 133.28 (CH) | 6.24 (m) |  | 131.0 (CH) | 6.22 |
| 20 | 132.86 (CH) | 6.31 (m) |  | 128.8^b^ (CH) | 6.30 |
| 21 | 133.35 (CH) | 6.32 (m) |  | 131.0^b^ (CH) | 6.30 |
| 22 | 131.51 (CH) | 6.19 (m) |  | 132.0^b^ (CH) | 6.30 |
| 23 | 133.57 (CH) | 6.28 (m) |  | 132.7^b^ (CH) | 6.30 |
| 24 | 132.93 (CH) | 6.13 (dd, J= 14.5 & 11.2 Hz) |  | 132.8^b^ (CH) | 6.30 |
| 25 | 130.93 (CH) | 5.80 (m) |  | 134.8 (CH) | 5.98 |
| 26 | 37.94 (CH_2_) | A: 2.24 (m)  B: 2.29 (m) |  | 73.0 (CH) | 3.94 |
| 27 | 70.31 (CH) | 4.97 (m) |  | 72.6 (CH) | 4.60 |
| 28 | 20.31 (CH_3_) | 1.17 (d, J=6.30 Hz) |  | 17.0 (CH_3_) | 1.18 |
| 29 | 10.93 (CH_3_) | 1.68 (s br) |  | 10.0 (CH_3_) | 1.67 |
| 1´ | 70.47 (CH) | 3.72 (m) |  | 69.5 (CH) | 3.66 |
| 2´ | 34.98 (CH_2_) | A: 1.31 (m)  B: 1.26 (m) |  | 33.6 (CH_2_) | A: 1.34  B: 1.25 |
| 3´ | 24.95 (CH_2_) | A: 1.44 (m)  B: 1.26 (m) |  | 30.6 (CH_2_) | A: 1.22  B: 1.18 |
| 4´ | 31.67 (CH_2_) | A: 1.25 (m)  B: 1.19 (m) |  | 24.0 (CH_2_) | A: 1.42  B: 1.24 |
| 5´ | 22.49 (CH_2_) | 1.25 (m) |  | 21.3 (CH_2_) | 1.23 |
| 6´ | 14.13 (CH_3_) | 0.86 (t, J=6.8 Hz) |  | 12.9 (CH_3_) | 0.83 |

^a^ Resonances can be interchanged.

Positions are labelled according to their number in the polyketide backbone (Fig. 5)
