# Supplementary material for: Functional analysis of filipin tailoring genes from Streptomyces filipinensis reveals alternative routes in filipin III biosynthesis and yields bioactive derivatives
Source: Microb Cell Fact. 2015 Aug 7;14:114. doi: 10.1186/s12934-015-0307-4 (PMC4527110; doi:10.1186/s12934-015-0307-4)
Supplement: Supplementary file 4 [file 12934_2015_307_MOESM4_ESM.docx]

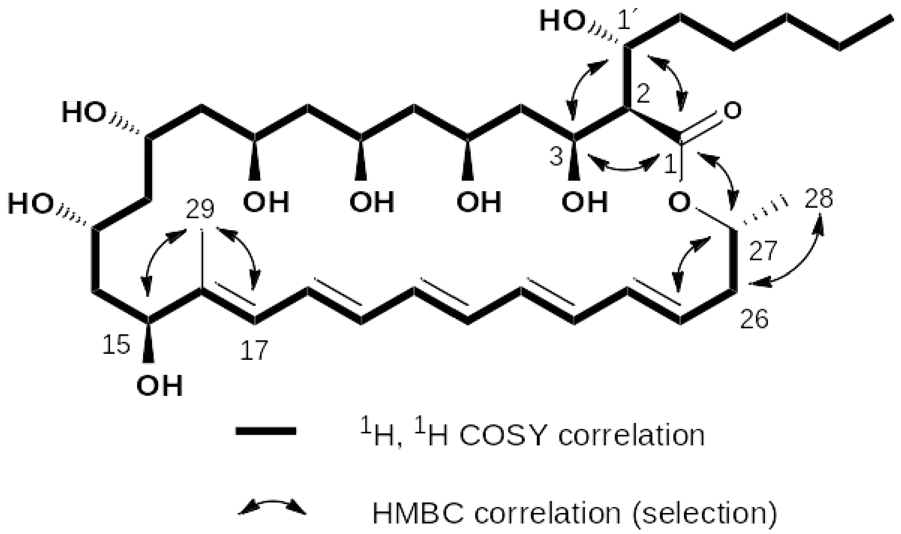
HO
1´
2
O
HO
3
1
OH
OH
OH
OH
O
28
HO
29
27
26
15
17
OH
1H, 1H COSY correlation
HMBC correlation (selection)
